# A multi-institutional survey of the quality of life after treatment for uterine cervical cancer: a comparison between radical radiotherapy and surgery in Japan

**DOI:** 10.1093/jrr/rraa107

**Published:** 2021-01-07

**Authors:** Yuko Kaneyasu, Hisaya Fujiwara, Tetsuo Nishimura, Hideyuki Sakurai, Tomoko Kazumoto, Hitoshi Ikushima, Takashi Uno, Sunao Tokumaru, Yoko Harima, Hiromichi Gomi, Takafumi Toita, Midori Kita, Shin-ei Noda, Takeo Takahashi, Shingo Kato, Ayako Ohkawa, Akiko Tozawa-Ono, Hiroki Ushijima, Yoko Hasumi, Yasuyuki Hirashima, Yuzuru Niibe, Tomio Nakagawa, Tomoyuki Akita, Junko Tanaka, Tatsuya Ohno, the Working Group of the Gynecological Tumor Committee of the Japanese Radiation Oncology Study Group (JROSG)

**Affiliations:** Department of Radiation Oncology, National Hospital Organization Fukuyama Medical Center, Hiroshima, Japan; Department of Radiation Oncology, Graduate School of Biomedical Sciences, Hiroshima University, Hiroshima, Japan; Department of Obstetrics and Gynecology, Chugoku Rosai Hospital, Hiroshima, Japan; Department of Obstetrics and Gynecology, Graduate School of Biomedical Sciences, Hiroshima University, Hiroshima, Japan; Radiation and Proton Therapy Center, Shizuoka Cancer Center, Shizuoka, Japan; Department of Radiation Oncology, University of Tsukuba, Ibaraki, Japan; Department of Radiation Oncology, Fukaya Red Cross Hospital, Saitama, Japan; Department of Radiation Oncology, Saitama Cancer Center, Saitama, Japan; Department of Therapeutic Radiology, Tokushima University Graduate School, Tokushima, Japan; Department of Diagnostic Radiology and Radiation Oncology, Chiba University Graduate School of Medicine, Chiba, Japan; Hyogo Ion Beam Medical Center, Hyogo, Japan; Department of Radiology, Saga University, Saga, Japan; Department of Radiology, Kansai Medical University, Osaka, Japan; Radiation Oncology Center, St. Marianna University School of Medicine Hospital, Kanagawa, Japan; Radiation Therapy Center, Okinawa Chubu Hospital, Okinawa, Japan; Department of Radiology, Graduate School of Medical Science, University of the Ryukyus, Okinawa, Japan; Department of Radiology, Tokyo Metropolitan Tama Medical Center, Tokyo, Japan; Department of Radiation Oncology, Saitama Medical University International Medical Center, Saitama, Japan; Department of Radiation Oncology, Gunma University Graduate School of Medicine, Gunma, Japan; Department of Radiation Oncology, Saitama Medical University Saitama Medical Center, Saitama, Japan; Department of Radiation Oncology, Saitama Medical University International Medical Center, Saitama, Japan; Department of Radiation Oncology, University of Tsukuba, Ibaraki, Japan; Department of Radiation Oncology, National Hospital Organization Mito Medical Center, Ibaraki, Japan; Department of Gynecology, St. Marianna University School of Medicine, Toyoko Hospital, Tokyo, Japan; Department of Radiation Oncology, Saitama Cancer Center, Saitama, Japan; Department of Obstetrics and Gynecology, Mitsui Memorial Hospital, Tokyo, Japan; Department of Gynecology, Saitama Cancer Center, Saitama, Japan; Division of Gynecology, Shizuoka Cancer Center, Shizuoka, Japan; Department of Public Health, Kurume University School of Medtioicine, Fukuoka, Japan; Department of Radiation Oncology, National Hospital Organization Fukuyama Medical Center, Hiroshima, Japan; Department of Epidemiology, Infectious Disease Control and Prevention, Graduate school of Biomedical and Health Sciences, Hiroshima University, Hiroshima, Japan; Department of Epidemiology, Infectious Disease Control and Prevention, Graduate school of Biomedical and Health Sciences, Hiroshima University, Hiroshima, Japan; Department of Radiation Oncology, Gunma University Graduate School of Medicine, Gunma, Japan

**Keywords:** uterine cervical cancer, radiotherapy, surgery, quality of life, questionnaire, sexuality

## Abstract

This study aimed to research the post-treatment quality of life (QOL) between radiotherapy (RT)- and operation (OP)-treated early cervical cancer survivors, using separate questionnaires for physicians and patients. We administered an observational questionnaire to patients aged 20–70 years old with Stages IB1–IIB cervical cancer who had undergone RT or OP and without recurrence as outpatients for ≥6 months after treatment. We divided 100 registered patients equally into two treatment groups (*n* = 50 each). The average age was 53 and 44 years in the RT and OP groups, respectively. The RT group included 34 and 66% Stage I and II patients, respectively, whereas the OP group included 66 and 34% Stage I and II patients, respectively. The OP group included 58% of patients with postoperative RT. Combination chemotherapy was performed in 84 and 48% of patients in the RT and OP groups, respectively. On the physicians’ questionnaire, we observed significant differences in bone marrow suppression (RT) and leg edema (OP). On the patients’ questionnaire, significantly more patients had dysuria and leg edema in the OP group than in the RT group, and severe (Score 4–5) leg edema was significantly higher in the post-operative RT group than in the OP only group. The frequency of sexual intercourse decreased after treatment in both groups. On the patients’ questionnaire, there were no significant differences between the two groups regarding sexual activity. These findings are useful to patients and physicians for shared decision-making in treatment choices. The guidance of everyday life and health information including sexual life after treatment is important.

## INTRODUCTION

Radiotherapy (RT) results at early-stage uterine cervical cancer are comparable to those of surgery [1]. However, in Japan, surgery-preferring gynecologists are responsible for determining treatment policies, therefore, most patients with stages IB–IIB cervical cancer were indicated for radical hysterectomy (RH) until recently. Conversely, because the National Cancer Institute (NCI) alert [2] recommends concurrent chemoradiation therapy [CCRT] for locally advanced cervical cancer, patients with stages IB2–IIB disease who were previously indicated for surgery are also increasly indicated for CCRT in Japan [3]. In the 2016 annual report of the Committee on Gynecologic Oncology, ~90% (3737/4164) of patients with stage I disease (18% [659/3737] received postoperative RT) and 47% (846/1804) with stage II disease (50% [420/846] received postoperative RT) underwent surgery, and only 9% (386/4164) and 52% (934/1804) of patients with stage I and II disease received radical RT, respectively [3]. From 1975 to 2000, the age of cervical cancer patients peaked at >75 years; however, from 2004, it peaked in an earlier bracket at 35–44 years, indicating that the incidence of cervical cancer is increasing among young Japanese women [4].

Posttreatment quality of life (QOL) is an important factor to consider before patients undergo treatment for uterine cervical cancer. Those with early cervical cancer have more than one treatment option; it is thus important that they understand the post-treatment change in QOL for each modality. Although the change in QOL after treatment is one of the crucial deciding factors in treatment selection, relevant information is limited because very few studies on this issue have been conducted in Japan [5, 6]. The long-term survival of young patients with cervical cancer highlights the importance of the late adverse events of treatment, particularly considering the increasing number of younger patients [2]. It is important to survey long-term survivors to understand the real-world situation with respect to adverse events associated with treatment and to determine the effects of different treatment approaches on QOL. Thus, the aim of the present study was to evaluate the incidence of adverse events and compare the differences in QOL between cervical cancer patients who underwent RT and radical operation (OP). These findings might help patients in the selection of treatment modalities. We present a multi-institutional study conducted by the Japanese Radiation Oncology Study Group (JROSG) gynecologic cancer committee.

## MATERIALS AND METHODS

### Patients

The present study was approved by the Institutional Review Boards (IRB) of all 12 institutions that participated in this study. The inclusion criteria were: (i) histologically confirmed FIGO Stages IB1–IIB cervical carcinoma; (ii) radical RT (combined external RT with intracavitary brachytherapy) or RH with or without postoperative RT: with or without chemotherapy; (iii) age = 20–70 years; (iv) recurrence-free; (v) final treatment was 6 months prior; (vi) performance status (PS) score of 0 or 1; (vii) could read and understand the questionnaire; (viii) no serious organ dysfunction and no psychological disease; and (ix) provision of written informed consent. The exclusion criteria were: (i) cervical stump cancer; (ii) use of conization or laser ablation technique and any other surgery except for RH; (iii) active double cancer patients who were treated and did not develop a recurrence within 5 years of treatment completion; and (iv) were judged unsuitable for inclusion in the study by a physician. We considered that PS ≤ 1 (0 or 1) is appropriate for comparing the RT group with the OP group because we sometimes encounter patients with PS ≥2 in the RT group, on the other hand surgery is usually difficult for patients with PS ≥2. The planned number of patients required for this study was 50 patients each in the RT and OP groups, total 100 patients.

At each institution, consecutive patients who met the eligibility criteria and obtained consent from the physician were enrolled between the January 2012 and April 2014. Patients in the OP group had undergone surgery between 22 August 1990 and 25 September 2009, and patients in the RT group had undergone radiotherapy between 12 December 1998 and 12 November 2008.

We conducted the questionnaire survey only once. The timing of evaluation after treatment differs for each patient. Therefore, after the evaluation, how the patient’s quality of life has changed is unknown except for items of dysuria described below from patients’ questionnaires.

### Patient recruitment

The recruitment procedure for this study varied by institution: in some institutions, the radiation oncologists asked the gynecologists to recruit all OP patients, while in others if the patients received post-operative RT the radiation oncologists were in charge of patient recruitment. The 21 patients who were treated with surgery alone without post-operative RT in the OP group were directly recruited by gynecologists (14 patients from 4 institutions), or referred by gynecologist to the outpatient department of radiation therapy, or the radiation oncologist went to the outpatient department of gynecology to recruit the patient (7 patients from 2 institutions). Radiation oncologists alone recruited and assessed both RT patients and OP patients in 7 institutions (in one institution only RT patients were recruited), of which in 6 institutions, radiation oncologists also recruited and assessed postoperative RT patients. In 3 institutions, patient recruitment was a joint effort between the radiation oncologists and gynecologists In 1 institution, radiation oncologist referred to the gynecologist for both recruitment and assessment of RT and OP patients. No patient was recruited in 1 institution. Hence, patients were recruited jointly by radiation oncologists and gynecologists in 4 institutions.

Overall 21 physicians including 14 radiation oncologists and 7 gynecologists cooperated in recruiting the patients and filling out the questionnaires. A total of 78 patients were recruited by radiation oncologists and 22 were recruited by gynecologists. Of the 50 RT patients, 49 were recruited by radiation oncologists and 1 patient was recruited by a gynecologist, and in the 50 OP patients, 29 were recruited by radiation oncologists and 21 by gynecologists.

Overall, 55 patients were recruited by the same physicians (23 RT patients and 32 OP patients), of which 49 patients (22 RT patients and 27 OP patients) were recruited by 5 radiation oncologists and the rest (1 RT, 5 OP) by a gynecologist. Of the remaining 45 patients, 29 patients (27 RT patients, 2 OP patients) were recruited by 8 radiation oncologists and the other 16 patients (OP patients only) by 6 gynecologists.

### QOL assessment

We assessed the patients’ QOL using The European Organization for Research and Treatment of Cancer (EORTC) Quality-of-Life questionnaire (QLQ)–Cervical Cancer Module (CX24) [7]. We obtained permission from the EORTC to translate and validate the QLQ–CX 24 for use in the Japanese population. Concurrently, while preparing the Japanese version of the QLQ–CX 24, our working group considered original questionnaires based on the literature, guidelines and clinical experience to ensure that the QOL was evaluated according to actual clinical practice in Japan.

We found that questions on treatment-specific adverse events and sexual life were not identified in the preparation of the Japanese version of EORTC QLQ–CX 24. Thus, we agreed to develop a questionnaire consistent with Japanese culture and lifestyle as follows.

We asked the patients about their surgical wound, included diarrhea and constipation as separate questions, and added a question regarding hematuria as a late adverse event of RT. Since dysuria may change immediately after treatment and over time, we asked for the most severe symptom immediately after treatment and then whether it improved. We evaluated abdominal pain using five grades: (i) slight pain; (ii) light pain; (iii) moderate pain; (iv) strong pain; (v) very intense pain, because it was subjective to each patient. With respect to sexual life, we asked whether the patients had a partner (Q21). In the EORTC QLQ–CX 24, sexual life during the past 4 weeks post-treatment is assessed; however, because Japanese sexual life is generally considered to be less active than that of the Westerners [8], we set this period at 1 year. We added a question on bleeding in addition to pain as a possible concern during sexual intercourse (Q23). Regarding the changes in sexual activity after treatment (Q32–45), we referred to a study by Sakurai *et al.* [9]. Finally, we created a 45-item QOL questionnaire for the present study (Table 1). A Japanese version of this QOL questionnaire is attached as Supplementary Table 1, see online supplementary material. We explained the background of the questionnaire development to the EORTC and obtained permission to use our modified version. The EORTC translation team leader instructed us to state that we referred to Greimel *et al*.’s paper [7]. A Japanese QOL questionnaire has been developed previously [10].

**Table 1:** Patient questionnaire

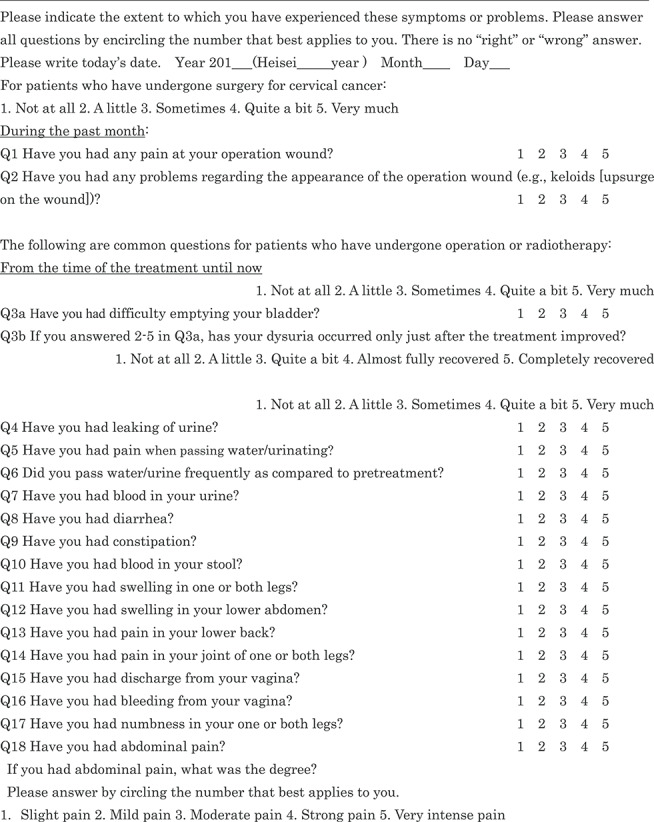
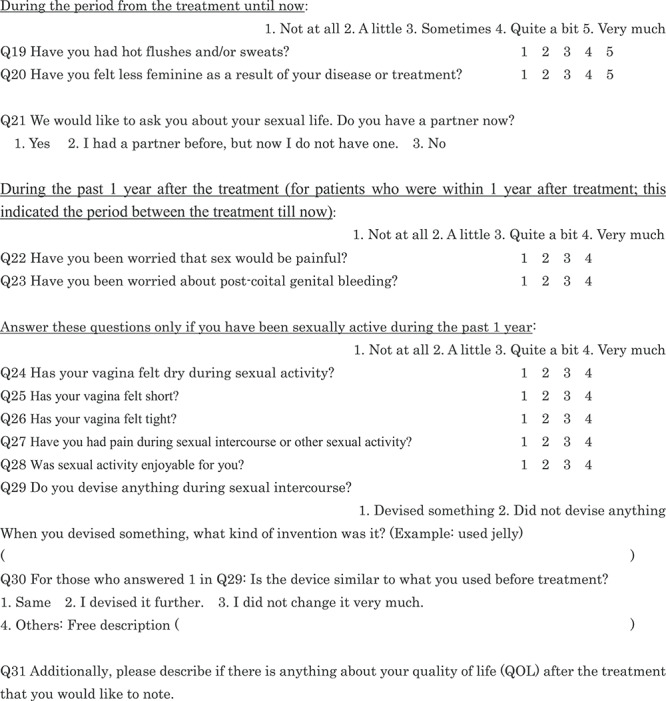
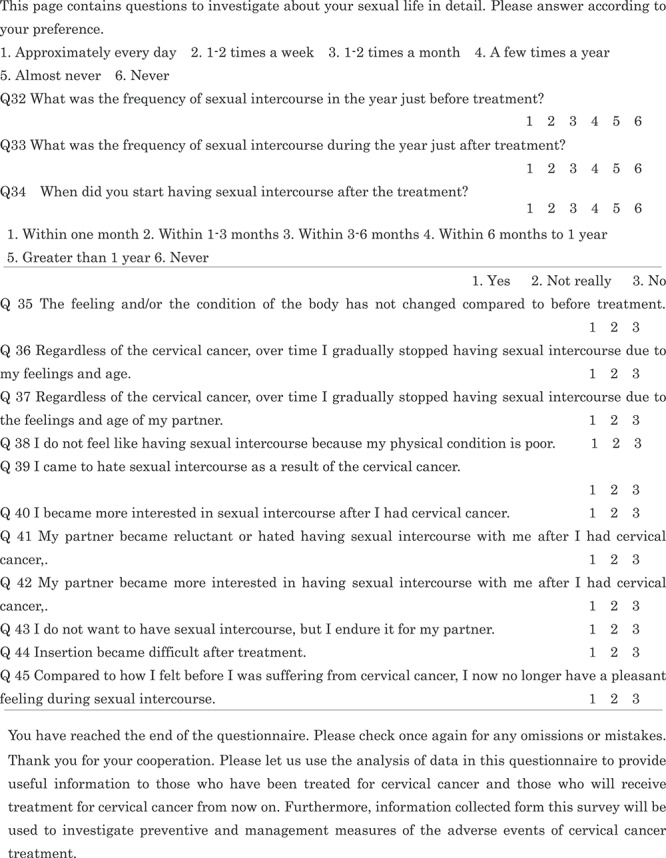

Our patients completed the Japanese version questionnaires in a private room and in compliance with the Japan Council for Quality Health Care standards.

### Collection of clinical information

The physicians confirmed that the patients met all the selection criteria and obtained written consent forms from each patient. The questionnaire we created for this study included 104 questions for physicians to collect information on (i) patient characteristics, (ii) treatment methods (radiation therapy, surgery, postoperative radiotherapy, combination chemotherapy), (iii) recurrences, (iv) adverse events, and (v) medical advice on their sexual life (Table 2). The Japanese version of this physicians’ questionnaire is provided as Supplementary Table 2, see online supplementary mateial.

**Table 2:** Physician questionnaire

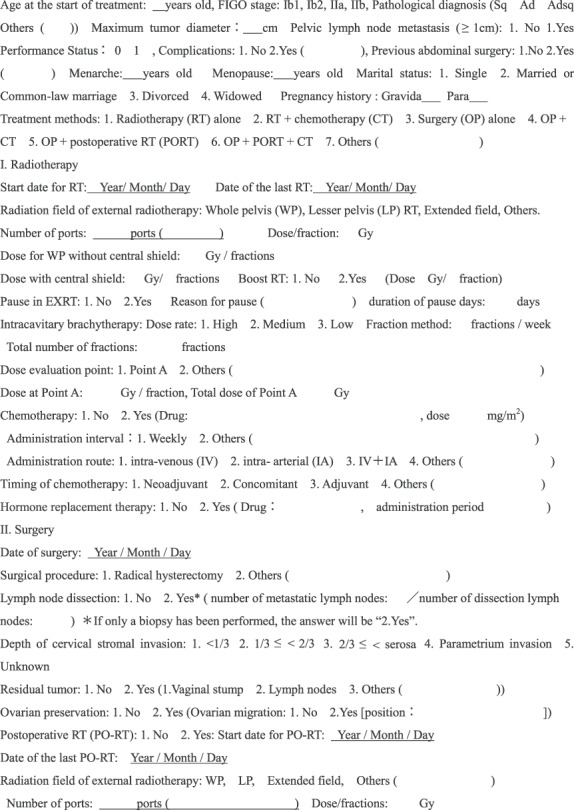
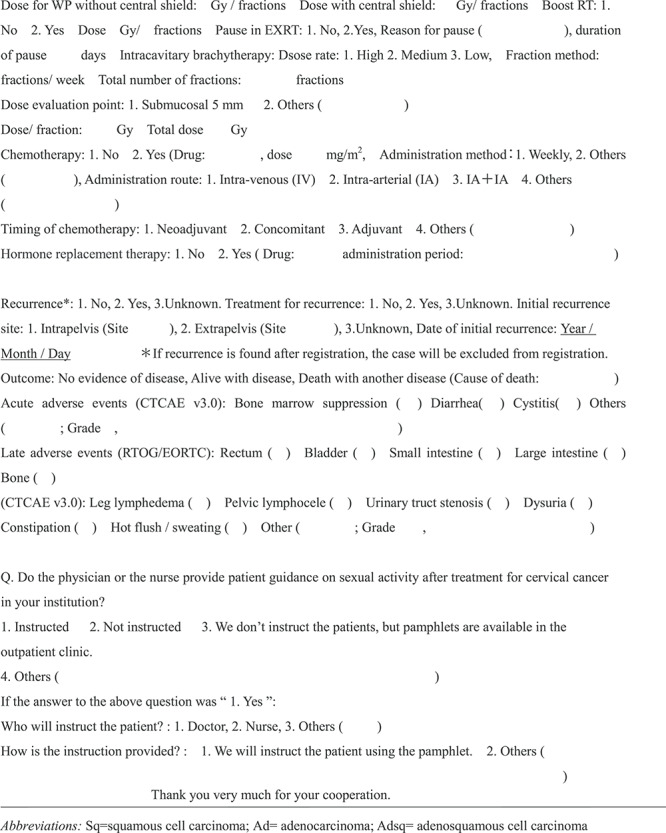

The physicians who participated in the study were 11 males and 10 females.

### After-treatment medical guidance on patients sexual activity by healthcare professionals

We confirmed whether the physician or a nurse provided guidance to the patients about sexual activity after the treatment of cervical cancer at each institution.

### Statistical analysis

We performed *t*-tests or Wilcoxon’s rank sum tests to compare continuous variables, and χ^2^-tests or Fisher’s exact tests were performed to compare categorical variables. We conducted a univariate analysis using χ^2^-tests or Fisher’s exact tests and a multivariate logistic regression analysis using a stepwise selection method to identify the factors associated with adverse events (14 variables) and QOL (45 variables) depending on the treatment technique (RT and OP). Factors that reached the 0.25 level of significance by stepwise procedure were included in the multivariate logistic regression analysis. All data were analyzed using JMP version 9 software (SAS Institute, Cary, NC, USA). For all analyses, *P* < 0.05 was considered statistically significant.

## RESULTS

### Patient characteristics

A total of 100 patients were registered from January 2012 to April 2014, with 50 patients each in the RT and the OP group (Table 3). There was no difference in the survey time from the treatment between the two groups (*P* = 0.346). In the RT group there were respectively 34 and 66% of patients with Stage I and II, while in OP group we had respectively 66 and 34. There were more stage I tumors in the OP group than in the RT group, and more stage II tumors in the RT group than in the OP group (*P* = 0.001). The patients in the OP group were younger than those in the RT group (*P* < 0.001). We have not investigated the body mass index of patients in both groups.

**Table 3:** Patients’ characteristics and treatment methods

		RT (*n* = 50)	OP (*n* = 50)	*P* –value
Age at treatment, years^a^		53 ± 12 (26–70)	44 ± 10 (26–67)	<0.001
Age at investigation, years^a^		56 ± 13 (27–76)	48 ± 10 (30–69)	0.001
Years from treatment, years^a^		3.2 ± 2.5	3.8 ± 3.7	0.346
Stage	I	17 (34%)	33 (66%)	
	II	33 (66%)	17 (34%)	0.001
Maximum tumor diameter (cm)^a,*^		4.4 ± 1.04	3.4 ± 1.25	0.004
Pelvic lymph nodes metastasis ≥1 cm				
Yes		19 (38%)	9 (18%)	
No		31 (62%)	41 (82%)	0.026
PS	0	43 (86%)	46 (92%)	
	1	7 (16%)	4 (8%)	NS^e^
Coexisting illness^b^				
Yes		9 (18%)	5 (10%)	
No		41 (82%)	45 (90%)	NS
Previous abdominal surgery^c^				
Yes		3(6%)	3(6%)	
No		47 (94%)	47(94%)	NS
Marriage history^d^				
Yes		47 (94%)	46 (92%)	
No		3 (6%)	4 (8%)	
Delivery history			
Yes		36 (73%)	44 (92%)	0.018
No		13 (27%)	4 (8%)	
Number of deliveries		2 (0–3)	2 (0–4)	
Postoperative RT (PO-RT)		—	29 (58%)	
PO-RT alone		—	11 (22%)	
PO-RT + chemotherapy		—	18 (36%)	
Surgery alone		—	14 (28%)	
Surgery + chemotherapy		—	7 (14%)	
Combination of chemotherapy		42 (84%)	25 (50%)	<0.001

^a^Means and standard deviations, otherwise numbers and proportions.

^b^E.g. diabetes mellitus, hypertension.

^c^E.g. appendectomy.

^d^Including common-law marriage.

^e^NS = not significant.

### Treatment

The RT group received a combination of external beam RT (EBRT) to the pelvic cavity and high dose rate intracavitary brachytherapy (HDR-ICBT). EBRT and HDR-ICBT were administered in accordance with the guidelines for RT included in *General Rules for Clinical and Pathological Study of Uterine Cervical Cancer in Japan* [11]. In the early part of EBRT, the median 30.4 Gy was delivered to the whole pelvis. Thereafter, the remaining the median 20 Gy was administered to the same whole-pelvic field with central shield. EBRT was given using the four-field box technique with 3D conformal radiation therapy (3D-CRT) for 37 patients (74%), and the parallel-opposed (anteroposterior–posteroanterior) technique for the other 13 patients (26%). Intensity modulated radiation therapy (IMRT) was not used. Eleven (22%) patients received boost irradiation with mean dose of 6.8 Gy, while 6 (12%) patients received extended field irradiation with whole pelvis and para-aortic region. The median number of HDR-ICBT fractions was 4, and the median dose at Point A/fraction was 6 Gy. The mean overall treatment time was 47.3 days. In 42 (84%) patients who received combined chemotherapy, cisplatin (CDDP) was used in 40 cases, mitomycin C in 6 cases and nedaplatin in 2 cases.

All of the patients in the OP group underwent RH, pelvic lymphadenectomy and bilateral salpingo-oophorectomy. The number of dissected pelvic lymph nodes was 7–120 (mean 38), and the number of metastatic pelvic lymph nodes was 0–20 (mean 1.08). One or both ovaries were preserved in 6 (12%) patients; all patients were in stage IB, and the mean patient age was 35 years (range, 27–48 years). In total, 29 (58%) patients received postoperative RT, 18 of whom also received chemotherapy. Of the 18 patients who received postoperative RT and chemotherapy, 5 received the neo-adjuvant chemotherapy (NAC) → OP→postoperative RT regimen and 13 received the OP→postoperative CCRT regimen. Meanwhile, 14 (28%) patients received surgery alone, and 7 (14%) patients received surgery + chemotherapy (2 patients received NAC, 4 received adjuvant chemotherapy and 1 received NAC and adjuvant chemotherapy). Of the 25 (50%) patients who were treated with combined chemotherapy, 12 were treated with CDDP. (Table 3).

### Adverse events according to the physicians’ questionnaire

The RTOG/EORTC classification lists the acute and late adverse events for RT patients, which do not apply to patients who have undergone surgery. So, we used the CTCAE classification to describe the late adverse events in OP patients.

Based on the physicians’ questionnaire, there were significantly more cases of bone marrow suppression (BMS) and diarrhea in the RT group than in the OP group as per the univariate analysis, and there were significantly more cases of BMS in the RT group than in the OP group in multivariate analysis (*P* = 0.013). On the other hand, there were significantly more cases of dysuria, leg edema, hot flush/sweating, constipation and pelvic lymphocele in the OP group than in the RT group in univariate analysis, and leg edema was significantly more common in the OP group than in the RT group in multivariate analysis (*P* = 0.006) (Table 4). In Table 4, all patients were evaluated for any adverse events other than urinary stenosis, which was not seen in any patient in the RT or OP group.

**Table 4:** Adverse events based on the physicians’ questionnaire^a^

	RT (*n* = 50)	OP (*n* = 50)	Univariate analysis			Multivariate analysis		
			OR^a^	95% CI	*P*-value	AOR	95% CI	*P*-value
BM suppression	39	21	0.2	0.1–0.5	<0.001	0.3	0.1–0.7	0.012
Diarrhea	36	23	0.3	0.1–0.8	0.008	0.4	0.1–1.1	0.064
Cystitis	9	6	0.6	0.2–1.9	0.400			
Dysuria	0	17	−		<0.001			
Constipation	0	4	−		0.041			
Pelvic lymphocele	0		−		0.041			
Hot flush/sweating	2	10	6.0	1.2–29.0	0.014	3.7	0.6–21.2	0.147
Rectum	4	1	0.2	0.03–2.2	0.169	0.0	0.01–1.26	0.073
Bladder	4	1	0.2	0.03–2.2	0.169			
Small intestine	3	3	1.0	0.2–5.2	1.000			
Large intestine	1	1	1.0	0.1–16.4	1.000			
Leg edema	1	15	4.9	1.5–16.2	0.005	6.4	1.7–24.0	0.006
Bone	2	1	0.5	0.04–5.6	0.558			

^a^OR = odds ratio (side effect occurred easily in the OP group if >1, and in the RT group if <1), AOR = adjusted odds ratio, CI = confidence interval, BM = bone marrow.

### Self-reported QOL according to the patients’ questionnaire

In total, 92% (92/100) of patients responded to the questionnaire (94% [47/50] and 90% [45/50] of patients in the RT and OP groups, respectively). As the questions were printed on both sides of the paper, some patients did not complete the form because they may have not noticed the back side.

In terms of subjective symptoms, there were significantly more patients with dysuria, constipation, leg edema, lower abdominal edema and hot flushes/sweating in the OP group than in the RT group in univariate analysis. In multivariate analysis, there were significantly more patients with leg edema and dysuria in the OP than in the RT group (Table 4).

### Dysuria and subsequent changes

Based on the physicians’ questionnaire, no cases of dysuria were reported in the RT group, while 34% of patients in the OP group experienced dysuria (Table 4, 0% [0/50] vs 34% [17/50], univariate analysis, *P* < 0.001). Based on the patients’ questionnaire, significantly more patients complained of dysuria in the OP group (44% [22/50]) than in the RT group (8.5% [4/47]) (Table 5, multivariate analysis, *P* = 0.022). Further, based on the responses provided in the patients’ questionnaire, patients who developed dysuria sometimes, quite a bit, or very much, in either group, improved quite a bit, almost fully, or completely recovered, in 68% (15/22: OP) and 50% (2/4: RT) of cases, with no significant differences between the patients in the OP or RT groups.

**Table 5:** Univariate and multivariate analysis of variable factors between the radiotherapy and operation group on the patients’ questionnaire

	Univariate analysis						Multivariate analysis		
Scale	Y/N^a^	RT	OP	OR^d^	95% CI	*P*-value	AOR	95% CI	*P*-value
**Clinical symptoms**									
***Genitourinary symptoms***									
Difficulty emptying the bladder	Y/N	4/43	22/28	8.4	2.6–27.1	< 0.001	4.6	1.3–19.1	0.022
Leaking of urine	Y/N	7/41	14/36	2.3	0.8–6.3	0.106	1.0	0.3–4.0	0.975
Pain when urinating	Y/N	4/46	1/48	0.2	0.02–2.2	0.176			
Increased frequency of urination	Y/N	12/38	8/39	0.6	0.2–1.77	0.396			
Hematuria	Y/N	2/48	2/46	1.0	0.1–7.7	0.967			
Vaginal discharge	Y/N	8/42	4/45	0.5	0.1–1.7	0.232			
Vaginal hemorrhage	Y/N	1/49	0/49	0.0		0.320			
***Gastrointestinal symptoms***									
Diarrhea	Y/N	17/33	18/30	1.6	0.5–2.7	0.718	0.6	0.2–2.0	0.446
Constipation	Y/N	15/35	25/24	2.4	1.1–5.5	0.033			
Blood in stools	Y/N	1/49	1/48	1.0	0.06–16.8	0.989			
***Pain***									
Lumbago	Y/N	14/36	15/34	1.0	0.4–2.7	0.775			
Pain of the inguinal region	Y/N	11/38	19/30	2.2	0.9–5.3	0.795			
Lower abdominal pain	Y/N	7/43	11/38	1.8	0.6–5.0	0.276	1.3	0.3–4.9	0.717
***Leg or lower abdominal lymphedema***									
Swelling in one or both legs	Y/N	3/47	17/32	8.3	2.3–30.8	<0.001	4.9	1.2–25.2	0.033
Lower abdominal edema	Y/N	1/40	9/40	11.0	1.3–90.7	0.007			
***Peripheral neuropathy***									
Tingling or numbness in feet	Y/N	9/39	11/38	1.3	0.5–3.4	0.653			
***Menopausal symptoms***									
Hot flushes and/or sweats	Y/N	10/37	19/26	2.7	1.1–6.8	0.031			
***Body image after suffering from a cancer***									
Feel less feminine as a result of disease or treatment	Y/N	7/39	8/37	1.2	0.4–3.7	0.742			
***Sexual partner existence***	Y/N	30/14	38/7	2.5	0.9–7.0	0.071			
***Sexual vaginal functioning***									
Vaginal dryness during sexual activity	Y/N	5/8	8/17	0.7	0.2–3.0	0.690			
Vaginal shortness	Y/N	1/12	6/19	3.8	0.4–35.5	0.219			
Vaginal tightness	Y/N	2/11	6/19	1.7	0.3–10.1	0.537			
Pain during sexual intercourse	Y/N	6/8	8/17	0.6	0.2–2.4	0.498			
***Sexual worry***
Worry about pain during sexual intercourse	Y/N	10/27	15/28	1.4	0.6–3.8	0.450			
Worry about post-coital genital bleeding	Y/N	6/31	11/33	1.8	0.6–5.2	0.334			
***Sexual activity, enjoyment and change***									
Enjoying sexual activity	N/Y	9/2	20/4	1.1	0.2–10	0.912		
Using something during sexual intercourse	Y/N	2/11	8/16	2.8	0.5–15.5	0.241			
Frequency of sex before the treatment^b^	Y/N	10/25	20/26	1.9	0.8–4.9	0.169			
Frequency of sex after the treatment^b^	Y/N	2/33	8/38	3.5	0.7–17.5	0.114			
The time you started having sex after treatmenct^c^	Y/N	7/26	17/27	2.3	0.8–6.6	0.102			
No change in the feelings and/or the condition of the body	Y/N	26/6	33/11	0.7	0.2–2.1	0.519			
I did not gradually do sex because of my feeling and age	Y/N	22/3	30/11	0.4	0.09–1.5	0.372			
I did not gradually do sex because of partner’s feeling and age	Y/N	20/4	30/12	0.5	0.1–1.8	0.278			
Because my physical condition is not good, I do not bring myself to do sex	Y/N	9/15	20/23	1.4	0.5–4.0	0.475			
Hate sexual intercourse as a result of cervical cancer.	Y/N	13/11	23/18	1.1	0.4–3.0	0.880			
I became more interested in sexual intercourse than previously	Y/N	4/20	7/36	1.0	0.3–3.7	0.967			
Partner declines or hates sexual intercourse after my treatment	Y/N	9/13	20/20	1.4	0.5–4.1	0.492			
Partner became more interested in having sexual intercourse	Y/N	4/17	6/34	0.8	0.2–3.0	0.685			
I do not want to have sexual inter-course, but I endure it for my partner	Y/N	6/10	14/29	0.8	0.2–2.7	0.721			
After treatment, insertion became difficult	Y/N	9/10	17/20	0.9	0.3–2.9	0.920			
No pleasant feeling at the time of sexual intercourse	Y/N	11/8	20/16	0.9	0.3–2.8	0.868			

^a^Y/N: Yes/No.

^b^Classified as Yes/No. Frequency greater than once a month: Yes (Y); frequency, less than once a month: No (N).

^c^Under 1 year: Y; interval of more than 1 year or never: N.

^d^OR = odds ratio (side effect occurred easily in the OP group if >1, and in the RT group if <1), CI = confidence interval, AOR = adjusted odds ratio.

On the other hand, postoperative dysuria was significantly more prevalent in patients in the postoperative RT group (48.3% [14/29]) than in patients in the OP alone group (14.3% [3/21]) (*P* = 0.009) based on the responses collected from the physicians’ questionnaire, while postoperative dysuria was not significantly different between the two groups (postoperative RT group (44.8% [13/29]) vs OP alone group (42.9% [9/21]) (*P* = 0.890)) based on the responses provided in the patient’ questionnaire.

### Leg edema in the OP group

**Fig. 1.**
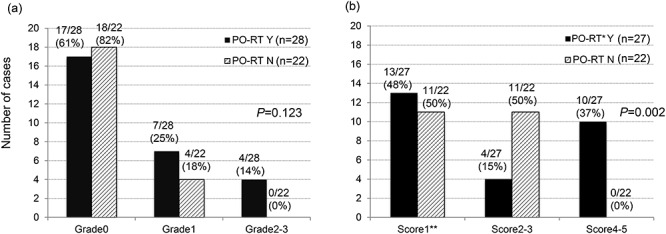
Degree of leg edema in those complaining of leg edema according to the presence or absence of postoperative RT (PO-RT) in the OP group on (**a**) the physicians’ and (**b**) the patients’ questionnaire. ^*^ One patient with PO-RT did not answer the question. Y: yes, N: no. Score1^**^, not at all; 2, a little; 3, sometimes; 4, almost always; 5, always.

Assessment of leg edema differs between the physician and the patient. As assessed by the physician, postoperative RT was not associated with a severe risk of leg edema (*P* = 0.123: Fig. 1a). However in the patients’ questionnaire, irreversible severe (Score 4–5) leg edema was significantly more observed in the postoperative RT group than in the RT only group (37% [10/27] vs 0% [0/22]; *P* = 0.002: Fig. 1b). There was a discrepancy between the physicians’ evaluation and the patient-reported outcome.

### Change in sexual activity

**Fig. 2.**
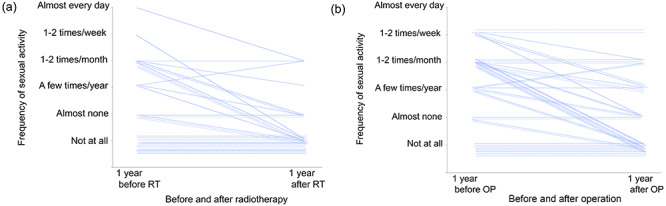
Change in frequency of sexual activity 1 year before or after (**a**) radiotherapy or (**b**) operation in patients with uterine cervical cancer.

In total, 88% of the patients answered detailed questions on sexual activity (Q32–45). There were no significant differences in sexual activity between the RT and the OP groups (Table 4). In total, 85% (85/100) of the patients responded to the question regarding the frequency and start time of sexual intercourse (Q32–34). Of these, 62% (53/85: RT, 52% [20/38]; OP, 70% [33/47]) were engaged in sexual activity within the past 1 year before treatment, while only 39% (33/85: RT, 26% [10/38]; OP, 47% [22/47]) were engaged in sexual activity within 1 year after treatment. This showed that the frequency of sexual intercourse at 1 year after treatment decreased significantly as compared to 1 year before treatment in both groups (overall: *P* = 0.001; RT group: *P =* 0.019; OP group: *P =* 0.021, Fig. 2).

### Medical guidance for patients regarding sexual activity

Following treatment, only 31% (31/100) of all patients received medical guidance from a physician or nurse. These included: 24% (12/50) and 38% (19/50) in the RT and the OP groups, respectively. Physicians or nurses provided direct guidance on sexual life to 4 and 17 patients in the RT and OP groups, respectively. Of the 21 physicians who participated in this study, 11 were males and 10 were females. However, the physician who filled out the questionnaire and the physician who gave the guidance regarding sexual intercourse after treatment to the patient were different for the OP alone group. For this reason, we researched the gender of the physicians who gave sexual advice. As shown in Table 2, the guidance items regarding sexual intercourse from medical staff to patients is: 1. Instructed, 2. Not instructed, 3. Pamphlet, 4. Others. We considered responses that were 1, 3, or 4 as instructed. Guidance rates for male doctors was 28.6% (18/63) and for female doctors 35.1% (13/37) (*P* = 0.493). There was no relationship between providing sexual activity guidance and the gender of the physician.

## DISCUSSION

Given the difference in treatment policies for RT and the surgical methods for uterine cervical cancer between Western countries and Japan, the post-RT or post-surgery QOL in Japan should be compared cautiously with that of Western countries.

In this study, we found significant differences in BMS in the RT group and leg edema in the OP group as assessed according to the physicians’ questionnaire. This finding was attributed to the significantly higher rate of combined chemotherapy in the RT group than in the OP group.

Postoperative lymphedema of the legs is a considerable problem due to RH, which includes complete pelvic lymph node dissection [12]. Many studies reported postoperative lymphedema of the legs [12–19], with the rate of lymphedema being higher in patients treated with OP and RT combined than in those treated with OP alone [19]. Further, one study reported that although emotional distress and QOL issues improved during the first 2 years after cervical cancer diagnosis, lymphedema and menopausal symptoms persisted [17]. In our study, leg edema was significantly more common in the OP group than in the RT group, according to both the physicians’ and the patients’ questionnaires. Meanwhile, the physician’s questionnaire showed no significant difference in the severity of postoperative lymphedema of the leg between the post-operative RT group and the OP only group. However, based on the patients’ subjective symptoms self-assessment, the degree of lymphedema of the leg in the OP group was significantly higher in patients with post-operative RT than that in the OP only group (Fig. 1).

In line with a previous study, we also observed a large gap between the patients’ subjective assessment of symptoms and physicians’ objective evaluation of lymphedema [5]. Despite the physicians’ assurance that the leg edema would cause minimal discomfort, most patients tend to consider leg edema as a major problem in performing the activities of daily living. Therefore, the self-reported patients’ QOL questionnaires in our study were considered very important.

Bladder, ano-rectal and sexual complications are common following RH for cervical cancer. In general, the incidence of temporary voiding dysfunction is higher following RH, while the incidence of urine storage dysfunction is higher following CCRT [20–22].

The major adverse event following RH for invasive cancer of the cervix is postoperative bladder dysfunction. Bladder dysfunction is a direct result of injury to the sensory and motor nerve supply to the detrusor muscle of the bladder [13]. Post-operative RT was associated with significantly more contracted and unstable bladder [13]. Butler-Manuel *et al.* compared QOL before and after OP and reported that urinary incontinence, particularly of urge incontinence, and voiding difficulties as well as tenesmus increased significantly after OP (*P* < 0.05 and *P* < 0.05, respectively) [20]. Like the findings of Katepratoom *et al.* [21], the incidence of difficulty in bladder emptying was significantly higher after OP as compared to RT in our study by both physicians’ (*P* < 0.001: univariate analysis) and patients’ (*P* = 0.022: multivariate analysis) questionnaires. In our study, the incidence of post-operative dysuria in the post-operative RT group was higher than in the OP alone group in the physicians’ questionnaire, but there was no difference between the two groups in the patients’ questionnaire. This was the opposite difference in assessment between physicians’ questionnaire and patients’ questionnaire to that for leg edema.

Rectal bleeding is a late adverse effect of RT [23,24]. Chronic adverse effects of intestinal RT can cause telangiectasis of the rectum and changes to the blood vessels of the rectal tissues [23]. However, we found no significant difference in rectal bleeding between the RT and OP groups in both the physicians’ (RT group: 8% [4/50], OP group: 2% [1/50], *P* = 0.073: multivariate analysis) and the patients’ (RT group: 2.0% [1/49], OP group: 2.1% [1/48], *P* = 0.989: univariate analysis) questionnaire in our study (Table 4 and 5). Diarrhea is a chronic symptom after RT [22, 25]. In our study, univariate analysis of the physicians’ questionnaire showed that diarrhea was significantly more frequent in the RT group (RT group: 72% [36/50], OP group: 46% [23/50], *P* = 0.008); however, there was no significant difference between the two groups as per the patients’ questionnaire (RT group: 39.4% [13/33], OP group: 60% [18/30], *P* = 0. 718). Hu *et al.* reported only minor differences in long-term QOL at least 2 years post-treatment between OP and RT patients, where pelvic neural dysfunction was significantly higher in the OP group, while intestinal dysfunction was higher in the RT group [24].

As sexual life differs between Japanese and Western people, a simple comparison is difficult. Japanese people are generally more reluctant to perform sexual activity [8] as compared to Westerners. Despite this, various reports have investigated post-treatment sexual activity in uterine cervical cancer patients. These reports show varying patterns of deterioration, compromise and improvement in sexual activity before and after various treatment modalities. Some authors have reported that cervical cancer survivors treated with RT had worse sexual function than the control group and those treated with RH and lymph node dissection [12, 18, 25–29]. Irradiated women faced more difficulty in becoming sexually aroused, attaining vaginal lubrication and achieving sexual satisfaction, and experienced significantly more pain during intercourse than those in the RH or control group [18, 30]. Meanwhile, Butler-Manuel *et al*. reported that 55% of patients considered that their sex life was worse after the surgery and 13% ceased having sexual activity [20]. Chronic fibrotic changes in pelvic tissue after RT create vaginal atrophy, which leads to persistent sexual and vaginal problems, such as dyspareunia and a lack of lubrication in cervical cancer patients [18, 27, 29]. These problems compromised their sexual activity and satisfaction. On the other hand, early diagnosis and treatment could facilitate a gradual return to a normal life and even an improvement in sexual activity in both those who undergo OP and CCRT + OP [16]. Compared to surgery alone, intracavitary RT, external RT, or both, in addition to or instead of surgery, had a small effect on the risk of reduced vaginal lubrication, shortness or inelasticity [31]. Kobayashi *et al.* [6] reported no significant differences in anxiety and depression scores among the three treatment modalities (RT, CCRT and OP + RT).

With respect to sexual activity, we found no statistically significant differences between the RT and OP groups based on the patients’ questionnaire. However, the frequency of sexual activity in both groups also decreased significantly after treatment as compared to before treatment, which was consistent with other reports [18,20].

In our study, only 31% of all patients (RT: 24%; OP: 38%) received guidance about post-treatment sexual activity from the medical staff; this low rate was attributed to busy outpatient clinics and lack of professional knowledge on sexual activities. Guidance rates of male doctors was 28.6% (18/63) and that of female doctors was 35.1% (13/37) (*P* = 0.493). There was no relationship between providing sexual activity guidance and the gender of the physician. Physicians are generally focused on monitoring relapse and late adverse events and do not have adequate time for consultations on the patients’ sexuality. Although sexuality is an important element of QOL, in Japan, there is insufficient support from healthcare workers on discussing sexuality. Further, it is difficult for patients to consult healthcare workers regarding sex [32]. Sexuality-related information in the context of adverse events after treatment should be provided to all patients, regardless of age or type of treatment [33]. Educating the medical staff on patient sexuality, providing information to patients and establishing a consultation desk is needed.

One of the causes of pain during sexual intercourse or during pelvic examination is dryness, vaginal adhesion due to RT and ovarian deficit symptoms due to oophorectomy. Vaginal pain can be improved by use of a vaginal dilator to prevent adhesion after RT or with jelly or mousse [34]. Preventing vaginal adhesion may lead to the early detection of cervical recurrence. The proportion of patients using a dilator after the initiation of RT was reported to decrease with time [35]. Training the medical support staff is also important. Books, pamphlets and lectures can be given to patients, partners and physicians to effectively provide more information on sexuality [32].

Our study has several limitations. First, this observational questionnaire survey study was not a prospective survey, and was conducted only once. However, there was no difference between the two groups with regard to the timing of the survey, so we evaluate that it is meaningful, even once. Second, since the questions were designed for a small number of patients in Japan, detailed analysis of every item was limited in between-group comparison. Third, post-operative irradiation was performed in 58% of the surgery group in this study. Since 67% (14/21) of the physicians who recruited patients were radiation oncologists, which was more than the 33% (7/21) of gynecologists, in this study more postoperative RT patients seemed to be recruited. Therefore, if we compare RT and OP alone, it seems to be biased. Fourth, the original QOL questionnaire used for this survey was developed by us. This QOL questionnaire, comprising a total of 45 items with disease-specific questions based on clinical practice in Japan, has not yet been validated. However, it is also being referenced in an ongoing Japanese clinical trial (JGOG1082). The usefulness of this QOL questionnaire should be further validated in prospective large-scale studies from now on. Future research requires a prospective design with long-term follow-up of QOL after treatment. To further develop our research, we are planning to release a pamphlet about the treatment of uterine cervical cancer.

## CONCLUSION

Post-treatment QOL change of RT and OP patients with early-stage cervical cancer were each characterized. Our findings will assist patients and physicians shared decision-making with respect to treatment choice. Healthcare professionals should provide patients with more guidance about coping with post-treatment changes in their QOL, including sexual life, irrespective of whether they undergo RT or OP.

## Supplementary Material

renamed_a4f9d_rraa107

renamed_bf8ba202012171735_rraa107
