# Erratum to: A multi-institutional survey of the quality of life after treatment for uterine cervical cancer: a comparison between radical radiotherapy and surgery in Japan

**DOI:** 10.1093/jrr/rrab007

**Published:** 2021-02-02

In the originally published version of this manuscript, several requested author amendments were inadvertently omitted prior to publishing. The publisher apologizes for the error and has since corrected these errors.

