# Supplementary material for: A multi-institutional survey of the quality of life after treatment for uterine cervical cancer: a comparison between radical radiotherapy and surgery in Japan
Source: J Radiat Res. 2021 Jan 7;62(2):269–84. doi: 10.1093/jrr/rraa107 (PMC7973450; doi:10.1093/jrr/rraa107)
Supplement: renamed_bf8ba202012171735_rraa107 [file renamed_bf8ba202012171735_rraa107.doc]

Supplementary Table 2. Physician Questionnaire

子宮頸癌治療後のQOL調査票(2) 主治医への調査用紙

201 年　　月　　日記入

施設名 　 記入者氏名： 　登録番号

治療開始時の年齢　　歳 FIGO病期： Ib1期、Ib2期、IIa期、IIb期

病理組織型：扁平上皮癌、腺癌、腺扁平上皮癌、その他( )

最大腫瘍径：　　　cm 、骨盤リンパ節転移（1cm以上）1.なし　　2.あり

PS：０、１ 合併症:1.なし　2.あり( 　　　　　 )

既往開腹術歴：**1.**なし　**2.**あり（　　　　　　　）初潮：　才、閉経：　才

結婚歴： **1.**未婚 **2.**既婚または事実婚 3. 離婚 4. 死別　妊娠•分娩歴：妊娠　　回、分娩　　回

治療内容

1.放射線単独　2.放射線と化学療法　3.手術単独　4.手術と化学療法　5.手術と術後照射

6.手術と術後照射と化学療法　7.その他（　　　　　　　　）

I.放射線療法

照射開始日：西暦　　　年　月　日　　照射終了日：西暦　　　年　月　日

外部照射照射野：全骨盤照射　　小骨盤照射　　拡大照射野 その他

門数　　　門(　　　　) 1回線量

中央遮蔽なし全(小)骨盤線量：　　　Gy/　　回、中央遮蔽あり骨盤線量：　　　Gy/　　回

ブースト照射の有無：1.なし　　2.あり 照射線量：　　Gy/　　回

外部照射休止の有無：1.なし　　2.あり 休止理由 　　　　　休止日数 日

腔内照射線量率：高•中•低　分割法：　回/週、回数：　回、線量評価点：1. A点 2.その他( 　　　　　　　　　　　) 、　一回A点線量 　 Gy、総A点線量 Gy

化学療法1.なし　2.あり(薬剤：　　　　、量　　　mg/m^2^、投与方法：weekly、その他

投与経路：静注、動注、静注＋動注、その他　　　　　　　　　　　　　　　　　　　　　)

化学療法併用時期：NAC、同時、Adjuvant、その他

ホルモン補充療法：1.なし　2.あり(薬剤：　　 　投与期間：　　　　　　 　　)

II.手術療法 　　　　手術日：西暦　　　年　月　日

Operative method: 1.広汎子宮全摘術　2.その他( 　　　　　　　 )

リンパ節郭清の有無：1. なし　2.あり* ( 転移リンパ節 個／ 郭清個数)

＊生検のみの場合は2.の、ありとする。

頸部間質浸潤の深さ：1. 1/3未満 2.1/3以上2/3未満　3. 2/3以上〜漿膜　4.子宮傍結合織浸潤　5.不明

術後遺残の有無：1.なし　2.あり（1. 腟断端 2. リンパ節 3.その他( 　　 )）

卵巣温存の有無：1. なし　2.あり (卵巣移動：1.なし2.あり(部位：　　　　　　　　　　))

術後照射： 1. なし　2.あり

照射開始日：西暦　　　年　月　日　　照射終了日：西暦　　　年　月　日

外部照射照射野：全骨盤照射　　小骨盤照射　　拡大照射野 その他

門数　　　門(　　　　) 1回線量

中央遮蔽なし全(小)骨盤線量：　　　Gy/　　回、中央遮蔽あり骨盤線量：　　　Gy/　　回

ブースト照射の有無：1.なし　　2.あり 照射線量：　　Gy/　　回

外部照射休止の有無：1.なし　　2.あり 休止理由 　　　　　休止日数 日

腔内照射線量率：高•中•低　　分割法：　回/週、回数：　回、　線量評価点：1. 粘膜下5mm 　2.その他( )、 一回線量 Gy、総線量 Gy

化学療法：1.なし　2.あり→薬剤：　　　　、量　　　mg/m^2^、投与方法：weekly、その他( )

投与経路：静注、動注、静注＋動注、その他（　　　　　　　　　　　　　　　　　　　）

化学療法併用時期：NAC、同時、Adjuvant、その他（　　　　　　　　　　　　　　）

ホルモン補充療法：1.なし　2.あり(薬剤：　　　投与期間：　　　　　　　 　)

再発*の有無: 1.なし　2.あり 3.不明　　再発への治療：1.なし　2.あり 3.不明

初回再発部位：1. 骨盤内(部位：　　 　　)　2.骨盤外 (部位：　　 　　)　 3.不明

初回再発確認日：西暦　　　年　月　日

最終観察日：西暦　　　　　年　月　日

＊登録後に再発が判明した場合は、解析から除外する。

転帰：無病生存、有病生存、原病生存、他病死(死因：　　　　　　　　　　　　　　　)

急性期有害事象グレード(CTCAE v3.0) :骨髄抑制( )、下痢( ) 、膀胱炎( )、その他：

( ：Grade , )

晩期有害事象グレード(RTOG/EORTC分類):直腸( )、膀胱( )、小腸 ( )、大腸( )、骨( )、(CTCAE v3.0) :下肢リンパ浮腫( )、骨盤内リンパ嚢胞( )、尿路狭窄( )、排尿障害( )、便秘( )、ほてりや発汗( )、その他( Grade 　, )

Q治療後の性生活に関して、医療サイドから患者へ指導を行っていますか？

1.指導している　2. 指導していない 3.指導していないが外来等にパンフレットを置いている4.その他（　　　　　　　　　　　　　　　　　　　　　　　　　　　　　　　　　　　　　）

上記で、1と回答された先生へ：指導は誰がしますか？　1.医師　2.看護師　3.その他( )

どのような指導をされてますか？ 1.パンフレットを使用して説明している

2.その他 ( )

ご協力ありがとうございました。
